# Automated scout-image-based estimation of contrast agent dosing: a deep learning approach

**DOI:** 10.1186/s12880-026-02331-1

**Published:** 2026-04-11

**Authors:** Robin Tibor Schirrmeister, Laetitia Taleb, Paul Friemel, Marco Reisert, Fabian Bamberg, Jakob Weiß, Alexander Rau

**Affiliations:** 1 Division of Medical Physics, Department of Diagnostic and Interventional Radiology, Medical Center - University of Freiburg, Faculty of Medicine – University of Freiburg, Freiburg, Germany; 2 Department of Diagnostic and Interventional Radiology, Medical Center - University of Freiburg, Faculty of Medicine – University of Freiburg, Freiburg, Germany; 3 Department of Stereotactic and Functional Neurosurgery, Medical Center - University of Freiburg, Faculty of Medicine – University of Freiburg, Freiburg, Germany; 4 Department of Neuroradiology, Medical Center - University of Freiburg, Faculty of Medicine – University of Freiburg, Freiburg, Germany

**Keywords:** Medical AI, Deep learning, Computed tomography, Contrast agent dosing

## Abstract

**Background:**

Optimal contrast agent dosing in computed tomography (CT) depends on accurate patient weight, yet manual measurements increase workload and self-reporting can introduce bias. We developed and tested a deep-learning-based algorithm to automate the approximation of contrast agent dosage directly from CT scout images.

**Methods:**

We retrospectively analyzed 817 patients undergoing thorax/abdomen CT. Prior to examination, patient weight was collected via manual scale measurements and self-reporting. We developed an EfficientNet convolutional neural network pipeline to estimate weight from scout images and used in-context learning and dataset distillation to analyze body-weight-informative CT features. The model was used in a browser-based user interface to provide dosing estimates for various contrast agent compounds.

**Results:**

Self-reported patient weights were statistically significantly lower than manual scale measurements (75.13 kg vs. 77.06 kg; *p* < 10^− 5^, Wilcoxon signed-rank test). In 5-fold cross-validation, the pipeline predicted patient weight with a mean absolute error (MAE) of 3.90 ± 0.20 kg. This error corresponds to a difference of roughly 4.48–11.70 ml of contrast agent, depending on the specific agent. Interpretability analysis confirmed that both larger anatomical shape and higher overall attenuation were the predictive features of body weight.

**Conclusions:**

This open-source deep learning pipeline enables automatic, accurate contrast agent dosing in routine CT workflows. The approach has the potential to improve patient safety and clinical efficiency by providing accurate weight estimates without requiring additional measurements or relying on potentially outdated records. Further validation on larger cohorts and across different clinical centers is required.

## Introduction

The administration of contrast agents is essential in various computed tomography (CT) examinations as this substantially improves the delineation of distinct anatomical structures, and their enhancement patterns facilitate the detection of pathologies [1]. Moreover, correct contrast agent dosing allows for reproducible imaging required for diagnosis and longitudinal follow-up [1]. Therefore, this constitutes an important step in the patients’ diagnostic journey. However, optimal dosage requires human interaction as optimal imaging results need patient-specific dosing of the contrast agent based on body weight, typically obtained through patient self-reporting or technician estimation, both of which may introduce bias and inaccuracies [2, 3]. Direct weight measurements using calibrated scales might provide the correct current weight, reduce bias and facilitate precise dosing but at the cost of substantially increasing the workload. Thus, it is not frequently integrated in the workflow. Deep learning algorithms have the potential to obtain patient-specific information from imaging data. A thus-derived body weight estimation might reduce workload and bias and improve reproducibility when determining optimal contrast agent doses for CT examinations.

Previous work has demonstrated the feasibility of deep-learning-based weight prediction from specific anatomical regions in CT scout images, which are required prior to the actual CT examination to estimate radiation dosage and adjust scan range. Studies have used deep neural networks to predict body weight from either thoracic or abdominal CT scout data separately. One study focused on pediatric patients [4] while another examined the general population [5]. However, these studies were limited to analyzing thoracic or abdominal scans in isolation and did not evaluate combined thoracic-abdominal scouts as a single input—which constitutes a very common acquisition range in clinical practice. Moreover, the applicability to clinical routine workflows is lacking, as no user interface for contrast agent dosing was proposed, though discussed as potential direction of future work in [5]. In addition, no in-depth analysis of the underlying predictive features was conducted, which may help to improve acceptability by users and identify challenging constellations that might require human interaction.

To address these limitations, we developed a deep-learning pipeline that estimates contrast agent dosage from CT scout images of a diverse, real-world dataset and provides an easy-to-use browser-based interface. For this, we retrospectively collected a clinical dataset of CT scout images and weight-scale measurements, and trained a deep network to predict body weight on these images, which are already collected during regular clinical CT examinations. We implemented this model in a browser-based interface that automatically estimates the optimal dosage for various contrast agents and can be easily integrated into clinical workflows. Additionally, we employed deep network interpretability methods to analyze the predictive features by distilling synthetic representative example training images for different body weights.

## Materials and methods

### Dataset

In this retrospective study, we included consecutive patients who underwent clinically indicated contrast-enhanced CT imaging of the thorax and/or abdomen at our tertiary referral center. All patients were scanned on either a NAEOTOM.alpha or Somatom.Edge (Siemens Healthineers, Erlangen, Germany) using routine protocols between 03/2021 and 07/2024. Scout data were obtained without any dose modulation and fixed 100 kV and 55 mAs (NAEOTOM.alpha) or 75 mAs (Somatom.Edge), respectively, with top tube position and the patient in supine position. Internal standard operating procedures advised technicians to ensure placement of the patient in the isocenter of the gantry. Body weight was both obtained via patients’ self reports and weight-scale measurements (Style Sense Comfort 300, SOEHNLE, Germany) by study staff as part of a quality assurance measurement immediately prior to the CT examination. Age and sex were obtained from clinical records. Pseudonymized data was assessed on 12 June 2023.

Patients were excluded if they were below 18 years of age or due to corrupted image quality of the scout, no weight could be reported by the patient, or if scale-based weighting was not feasible, e.g. due to immobility.

**Fig. 1:**
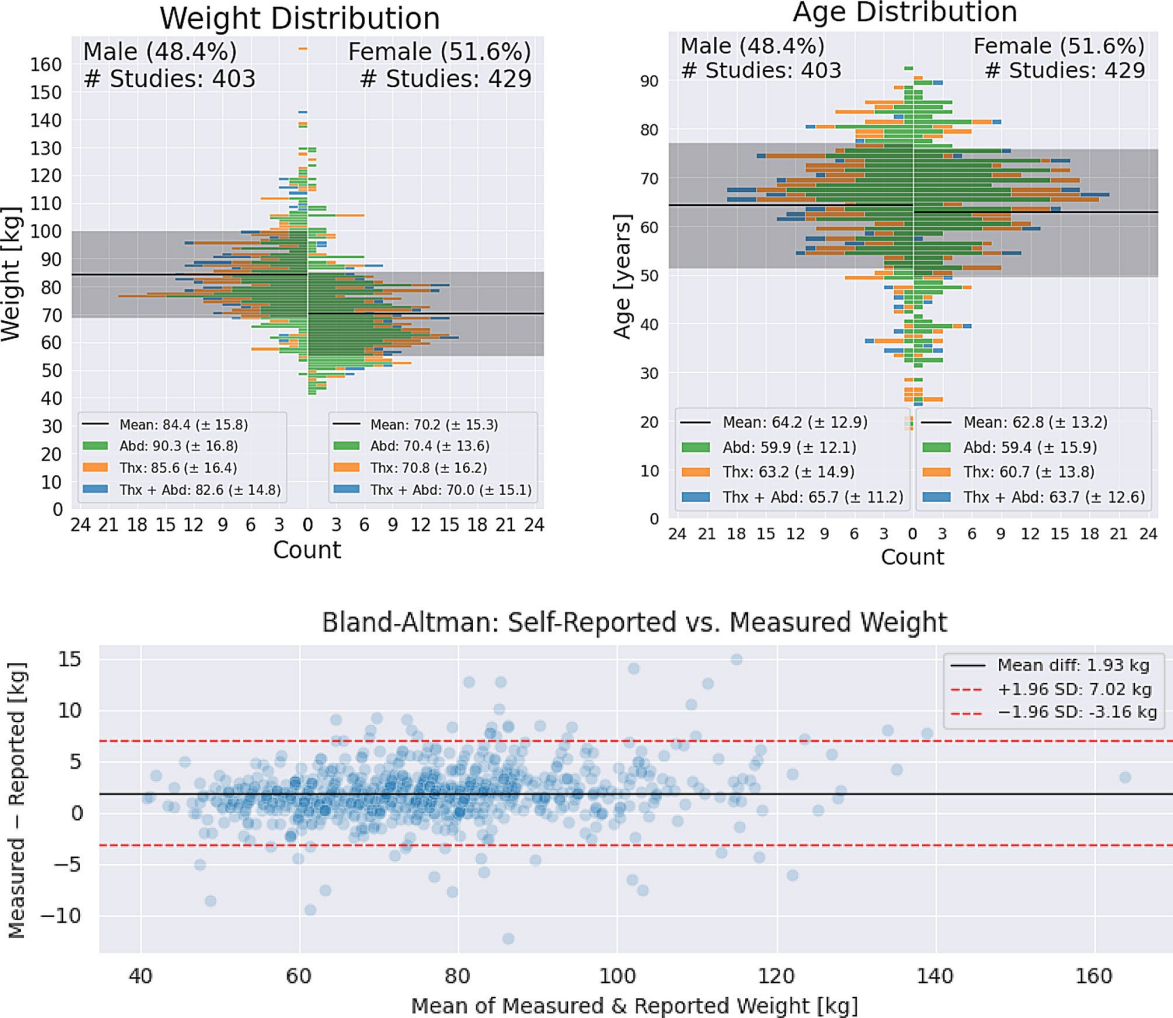
Distribution plots for weights and ages and Bland-Altman plot for reported and measured weight. Our cohort contains a large diversity of weights and ages for both sexes and the different anatomical regions. Distribution of body weights. Bars show the number of persons with a given body weight in our cohort, separated by abdominal, thoracic or thoracic-abdominal scans. Distribution of ages. Bars show the number of persons with a given chronological age in our cohort, separated by abdominal, thoracic or thoracic-abdominal scans. Bland-Altman plot of self-reported vs. measured weight. Each marker represents the mean (x-axis) as well as the difference (y-axis) of measured weight and self-reported weight for one person. Overall, the self-reported weights track the measured weights fairly closely, however, the measured weights were systematically larger than the self-reported weight

Our cohort contains a large diversity of weights and ages for both sexes and the different anatomical regions, see Fig. 1.

### Model development and testing

**Development**: For weight prediction from CT scout scans, we employed the widely-used EfficientNet architecture [6], specifically its EfficientNet-B1 variant, implemented through the MONAI library [7]. This architecture has demonstrated success in preliminary experiments and has previously been used for CT scout-based weight prediction [4]. We optimized the network parameters using AdamW with an L1-Loss function, using a learning rate of 1.5 × 10^− 4^ and weight decay of 10^− 5^. For data augmentation, we implemented a subset of techniques from nnUNet, namely random cropping, low-resolution simulation, Gaussian noise addition, Gaussian blurring, and random gamma transformation.

**Testing**: The model was tested via 5-fold cross-validation, with the training folds always containing 80% and the test folds containing the remaining 20% of the full dataset. We report overall results as well as results grouped by the different anatomical regions.

### Model interpretability

To gain insight into the predictive features that enable automatic body-weight predictions, we performed an interpretability analysis combining a variational autoencoder with in-context learning. The rationale for this approach is to move beyond local explanations (e.g., heatmaps) and instead synthesize a “distilled” dataset that represents the global visual features associated with weight variations.

### Synthetic data generation through dataset distillation

We used in-context learning to synthesize a small representative training dataset that can be visually inspected for recognizable features. In-context learning, for example with the TabPFN network [8], uses an entire training dataset as context to predict test data without any training or finetuning of the network parameters, analogous to how an LLM uses a language prompt as context to generate an answer without adjusting the LLM parameters. Crucially, this approach also enables the distillation of a smaller optimized synthetic training dataset that performs well as context to predict the original data. To achieve this, a randomly initialized synthetic training context dataset is iteratively updated with gradient descent to lower the prediction loss on the complete real training dataset, ultimately yielding a synthetic dataset that works well as a context to predict the real data. For a general explanation of this context-tuning method, see [9].

To apply TabPFN, we downsampled the images to 32 × 32 resolution and subsequently trained a variational autoencoder to further compress them into a 16-dimensional latent representation. The context-tuned latent representations are later decoded back to image space for visualization.

The synthetic pipeline was applied to the full training dataset without preselection of patient subgroups or poses, so that it accurately reflects our complete clinical population and avoids selection bias. We then visualized the entire synthetic training dataset to identify visual patterns predictive of body weight.

### Contribution to study objectives

This distillation process provides a compact, human-interpretable “gallery” of learnable weight-predictive features. If the distilled images show recognizable anatomical changes corresponding to weight increases (e.g., widening of the lateral body contours), this confirms that the relevant biological signals can be learned by the model on this data. This increases the clinical trustworthiness of the primary weight-prediction model by suggesting that the predictive signal underlying dosing recommendations is grounded in patient anatomy.

### Statistical analysis

We evaluated our results using two statistical approaches: a Wilcoxon signed-rank test to compare reported versus measured weights, and cross-validation to assess prediction accuracy. The Wilcoxon test determined if the differences between reported and measured weights were statistically significant. From the 5-fold cross-validation, we calculated both the average mean absolute deviation across folds and its standard error to quantify prediction performance.

## Results

### Participants

The cohort consisted of 533 thoracic, 70 abdominal and 229 thoracic-abdominal CT scout scans. Patients were between 18 and 92 years old, 403 male (48.8%) and 429 female (51.6%). The patients had a large variety of body weights as shown in Fig. 1. Thirty-eight patients had to be excluded due to corrupted image quality of the scout (*n* = 8), no weight could be reported by the patient (*n* = 2), or if scale-based weighing was not feasible (*n* = 28), e.g. due to immobility.

**Fig. 2:**
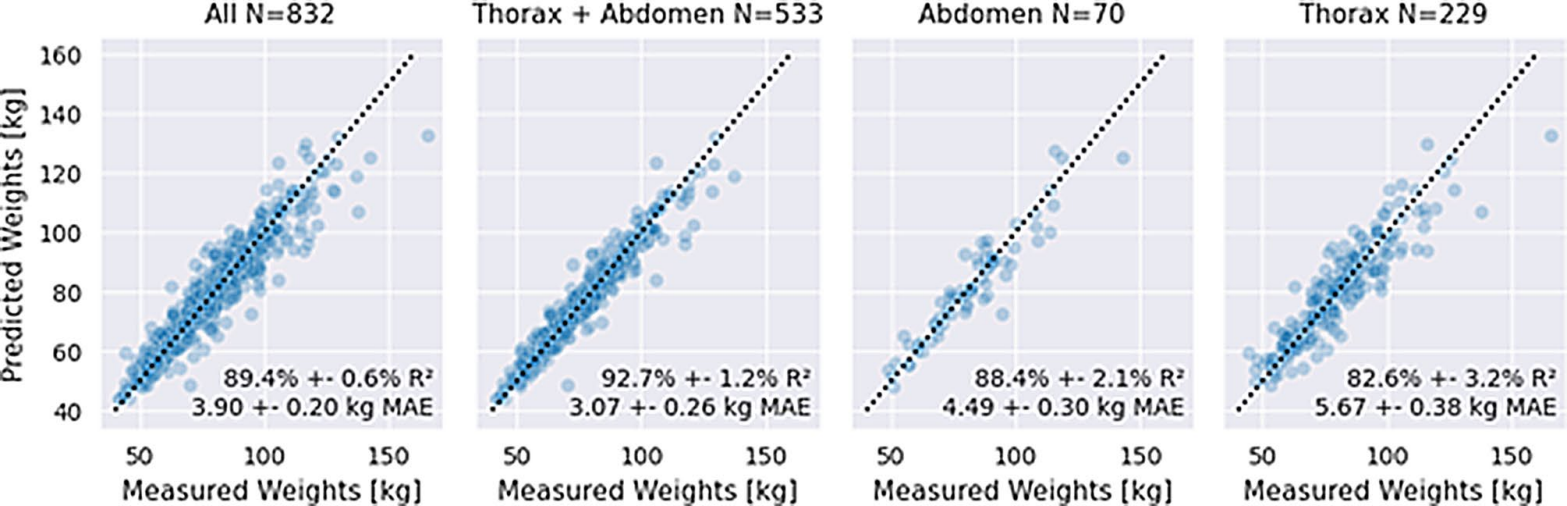
Model performance in CT-scout-based weight prediction. Each marker represents the measured and predicted weight for one person. Predictions for all regions have a lower mean absolute error (MAE) than 5.5 and Pearson-R^2^ correlations above 80%, thoracic-abdominal scans are predicted most accurately

The reported weights are reasonably accurate, yet systematically underestimate the measured weights as depicted in Fig. 2. After removing seven cases with apparent data entry errors (such as recorded height instead of weight), we found that self-reported weights were significantly lower than manual measurements (75.13 kg vs. 77.06 kg; *p* < 10^− 5^, Wilcoxon signed-rank test). With a mean absolute difference to the measured weights of 2.47 ± 0.06 kg across all studies, these self-reported weights provided a strong baseline for evaluating the prediction differences of our automated pipeline and the measured weights.

### Model testing

**Fig. 3:**
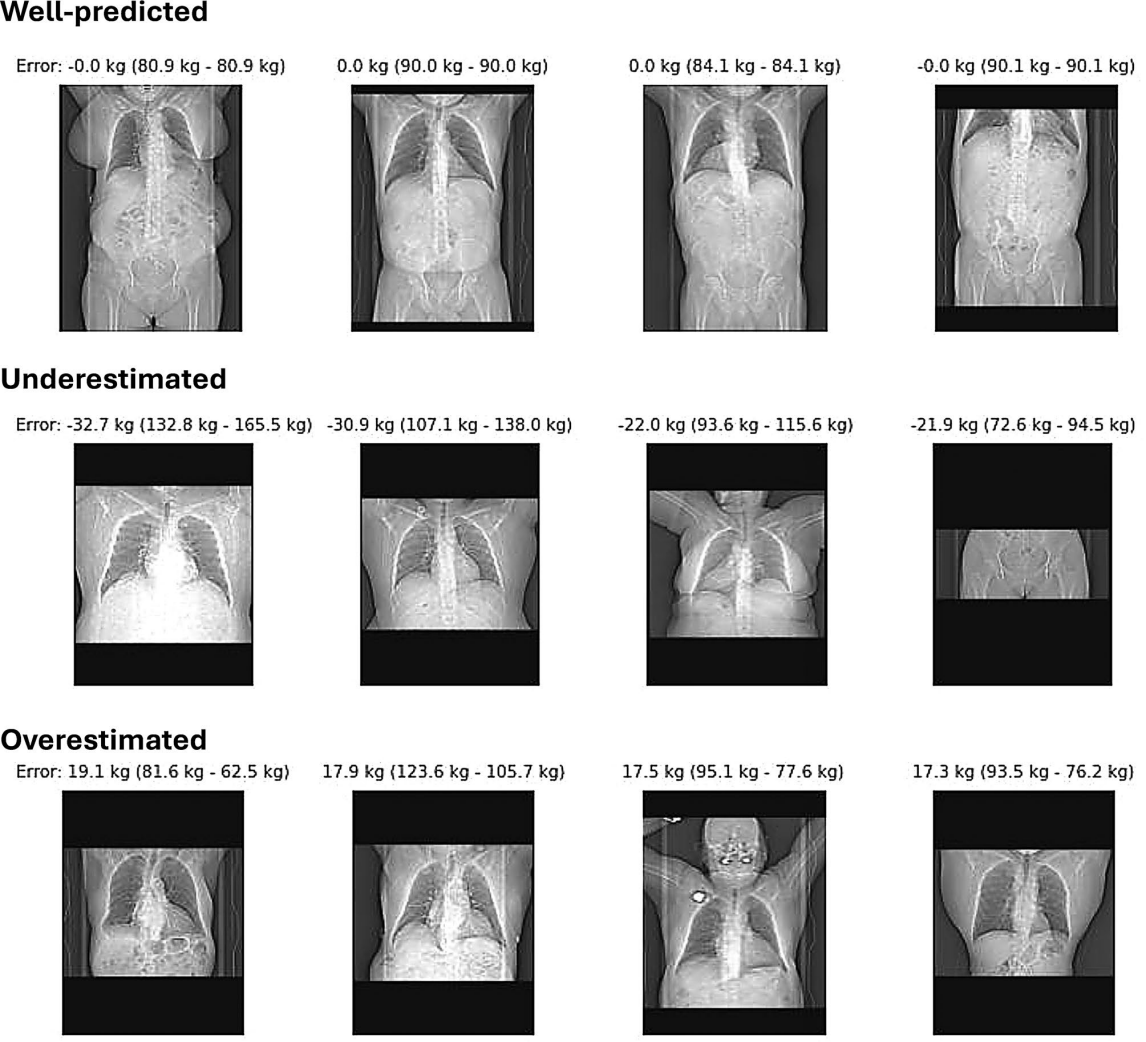
Examples of well-predicted, underestimated, and overestimated cases. Scans from a variety of regions appear in the well-predicted, underestimated, and overestimated categories, with no obvious patterns except for lower weights being more overestimated and higher weights more underestimated

In the whole dataset, using 5-fold cross-validation, we found an overall sufficient performance of automated weight prediction with a mean deviation of 3.90 ± 0.20 kg (mean and standard deviation across the 5 folds) of predicted and scale-measured body weight. Higher performance was noted in scans that encompassed the thorax and abdomen (3.07 ± 0.26 kg), while slightly lower performance was noted for scans of either the abdomen (4.49 ± 0.30 kg) or the thorax (5.67 ± 0.38 kg). The superior performance on thoracic-abdominal scans can likely be attributed to the larger body area providing more weight-relevant information. Notably, the model performed well on abdominal scans despite their limited representation in the dataset (<10% of total data). Exemplary cases of well-predicted as well as over- and underestimated weight are shown in Fig. 3.

Analysis of prediction errors revealed no clear patterns across anatomical regions, with well-predicted, underestimated, and overestimated cases distributed across all scan types.

**Fig. 4:**
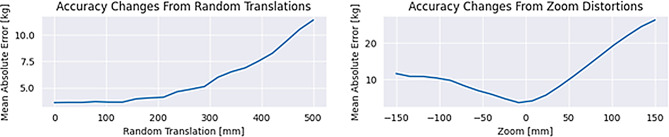
Model performance under simulated spatial perturbations. Left plot shows accuracy under random translations of fixed magnitudes, right plot shows accuracy under zoom distortions. Model is fairly robust to translations and more sensitive to zoom distortions

To obtain a rough estimate of model robustness to patient positioning variability, we evaluated model performance under simulated spatial perturbations of the scout images (see Fig. 4). Specifically, we applied vertical translations (from 0 to 500 mm) and isotropic zooming (corresponding to magnification changes from approximately −150 to + 150 mm off-centering along the beam axis, using the scanner’s source-to-isocenter distance of approximately 600 mm). These simulations represent simplified approximations of real positioning effects; in particular, the isotropic zoom does not capture the asymmetric magnification produced by divergent beam geometry, where the body side closer to the source is magnified more than the distal side. With these caveats, translations of up to approximately 100 mm had little effect on prediction accuracy, with gradual degradation beyond that range. Zoom perturbations led to a more rapid decrease in accuracy, indicating body silhouette area as a predictive feature.

### Model interpretability

**Fig. 5:**
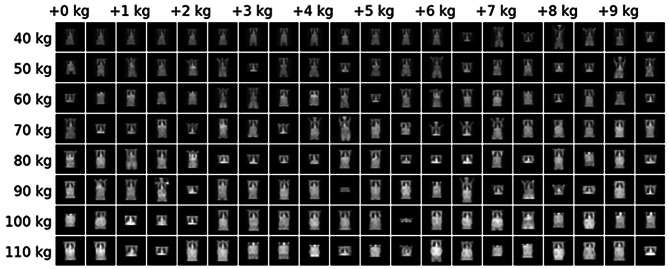
Selected CT scout images from lower to higher weights in kg. Input images are shown as they are supplied to the deep network during training. A large diversity of shapes is visible, with images with larger body weight having larger shapes and more attenuation

Visual analysis of the original CT scout scans downsampled to 64 × 64 and sorted by measured body weight revealed mixed patterns as seen in Fig. 5. While patients with higher body weight generally showed larger attenuations and anatomical shapes as expected, substantial variation existed among scans from patients of similar weights. This variability makes it challenging to identify which specific features are sufficient for robust predictions.

**Fig. 6:**
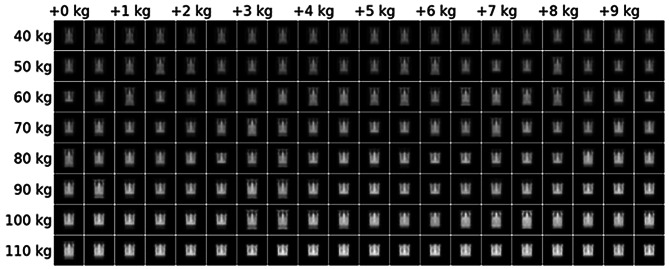
Synthetic distilled dataset from lower to higher weights in kg. Dataset was synthesized to perform well as an in-context training set and is sufficient to reach below 5 kg mean absolute error. Note the smooth progression from smaller anatomical shapes and less attenuation to larger shapes and stronger attenuation with increasing body weight

We used in-context learning combined with dataset distillation to generate a synthetic training dataset that yields high accuracy when used for learning the body weight prediction. Such a synthetic dataset can show general patterns informative for the task, going beyond traditional saliency-based methods explaining individual example predictions. The synthetic distilled training dataset in Fig. 6 shows a more consistent pattern from lower attenuation and smaller anatomical shape to higher attenuation and larger anatomical shape with increasing body weight. Using in-context learning with this distilled dataset still retains an average MAE below 5 kg, indicating that the contained information is enough to explain a substantial amount of the predictive features. Overall, this suggests that the expected relationship of attenuation and anatomical size with body weight contains sufficient information for accurate predictions. Hence, this analysis increases trust in the weight prediction pipeline as it demonstrates the presence of learnable features grounded in patient anatomy.

### Open-source web user interface

**Fig. 7:**
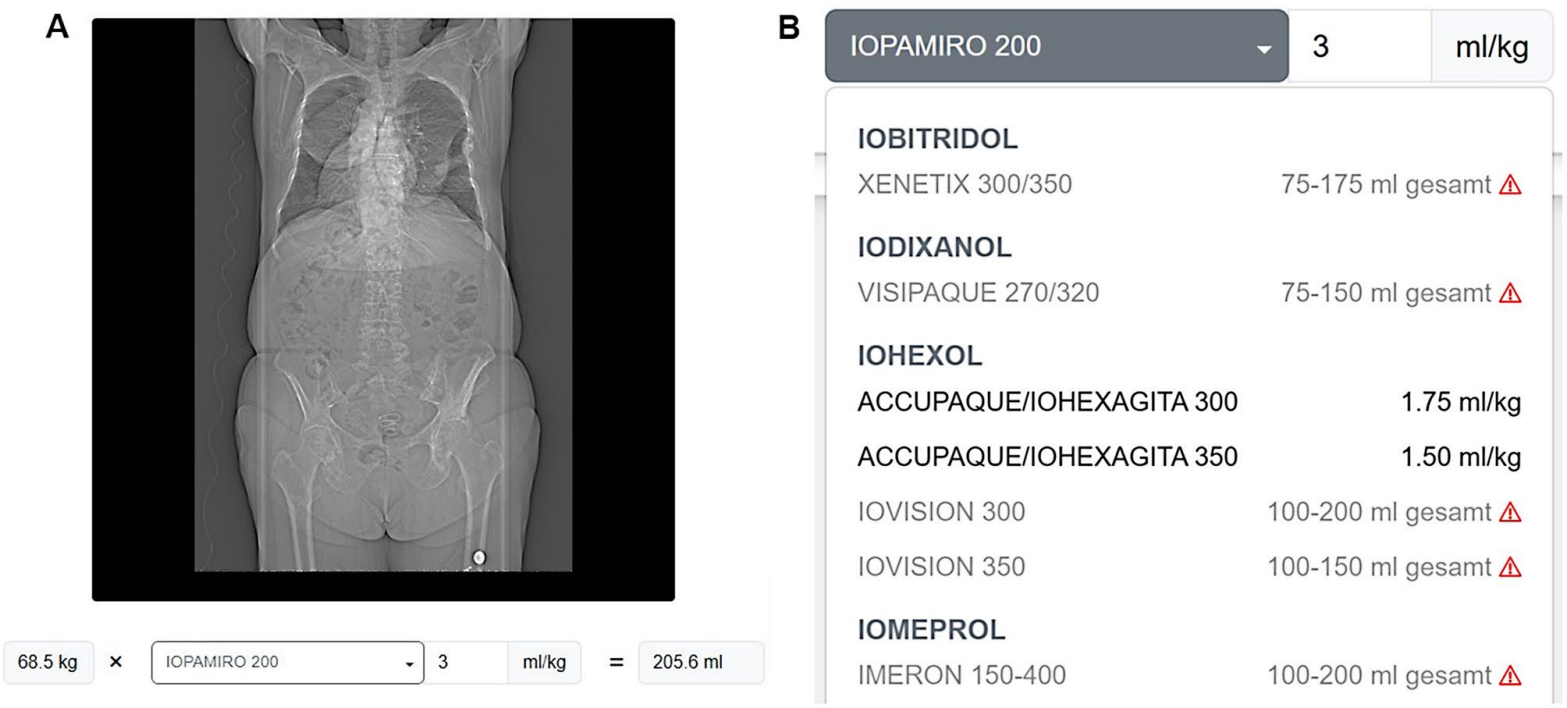
**A**) Image, predicted weight and suggested dosage. The user-provided scan is shown together with the predicted weight and the dosage specific to the contrast agent chosen by the user. **B**) contrast agent choice. Dropdown menu to choose between various contrast agents (subset shown here) to use their conversion factors from body weight to dosage. Some manufacturers only provide a total dosage for their agents. UI also allows for inputting a custom conversion factor directly

We developed a browser-based user interface that enables CT scout image upload and provides contrast-agent-specific dosage estimates using the hereby developed model. Users can input CT scout scans as NIFTI files and, as shown in Fig. 7, select contrast agents from a dropdown menu (Fig. 7B), with dosage suggestions calculated using either manufacturer-provided or custom conversion factors (Fig. 7A). The interface runs locally using ONNX Runtime [10], ensuring data privacy while delivering weight predictions in approximately one second on standard hardware (Intel Xeon CPU with 6 Cores at 3.5 GHz, no GPU). The tool is publicly available at https://tinyurl.com/ct-scout-weight. This open-source calculator was employed to translate the prediction error of body-weight to contrast agent volumes corresponding to a difference of 4.48–11.70 ml of contrast agent, depending on the specific agent (dosages obtained from the respective product information).

## Discussion

In this study, we present an end-to-end, scout-image-based workflow that (1) estimates patient body weight using a deep learning model trained on routinely acquired CT scout images and (2) translates the predicted weight into contrast agent dosing via a browser-based user interface. Because scouts are already acquired for essentially every CT examination, this approach targets a practical workflow bottleneck: obtaining an accurate, up-to-date body weight without adding manual steps, relying on outdated records, or introducing patient self-report bias. The overall prediction performance (mean absolute error around 4 kg in cross-validation) indicates that scout images contain sufficient information to approximate body weight with clinically relevant accuracy, and that this information can be extracted robustly across common thoracic, abdominal, and combined thoracoabdominal acquisition ranges.

Accurate weight is a central input to weight-adapted contrast dosing schemes. Our results demonstrate that self-reported weights were systematically lower than scale-based measurements. In routine practice, such underreporting may translate into underdosing and thereby potentially reduced vascular and parenchymal enhancement, particularly in examinations where consistent enhancement is essential for diagnostic confidence and longitudinal comparability. Automating weight estimation from scouts therefore offers a pragmatic route to reduce one avoidable source of dosing variability without changing acquisition protocols.

Our results align with the small body of published work on deep-learning-based weight estimation from CT scouts. Okabayashi et al. [11] reported MAEs of 2.75 kg (chest) and 4.77 kg (abdomen) on 2,350 adults using VGG16, while Demircioğlu et al. [4] achieved 4.25 kg on 889 pediatric patients. Our thoracic-abdominal MAE of 3.07 kg is competitive, approaching the chest-only benchmark from a nearly three-fold larger dataset.

The model tended to underestimate higher body weights more than lower body weights. This pattern is common in regression settings, where uncertainty and loss minimization can bias predictions toward the center of the training distribution [12]. In practice, this implies that the most relevant failure mode is not random noise but a mild compression of extreme values. From a dosing perspective, this matters because the clinical penalty of underestimating very high weights can be larger than small errors near the mean. Consequently, a conservative implementation should (a) allow easy manual override, (b) flag predictions near the extremes of the training distribution, and (c) consider calibration strategies that explicitly reduce bias at high weights (for example, loss reweighting, stratified sampling, or post-hoc correction based on residual trends).

We attempted to identify systematic patterns in failure cases, but mispredictions were distributed across acquisition regions without an obvious single artifact signature, and substantial appearance variation existed even among patients with similar measured weights. The synthetic dataset did not reveal a systematic pattern neither for misprediction, though the overall features of the model performance were comprehensible with a smooth progression from smaller anatomical silhouettes and lower overall attenuation to larger silhouettes and higher attenuation with increasing body weight.

A practical concern for any scout-based anthropometric estimation is patient positioning relative to gantry isocenter. Off-centering changes apparent magnification and attenuation pathways and could therefore influence both shape-derived and attenuation-derived cues used by the model [13, 14]. Our simulation-based robustness analysis suggests that the model tolerates moderate translational offsets but is more sensitive to magnification changes, which is expected given the role of apparent body silhouette area as a predictive feature. However, these simulations are simplified approximations; in particular, divergent beam geometry produces asymmetric rather than uniform magnification, and real off-centering may co-occur with other acquisition variability not captured here. In our setting, technicians were instructed to position patients at isocenter, but the degree of off-centering could not be quantified retrospectively and thus represents a limitation. Future prospective work should explicitly capture table height and lateral offset (where available) or use reconstructed localizer geometry metadata to quantify and potentially correct for positioning effects. Furthermore, tissue-equivalent phantoms might allow for further disentangling factors for improving model performance.

A key contribution of this work is not only the model but its translatability into clinical routine. For this, we provide an open-source online calculator that provides contrast agent-specific dosages after input of a scout. Here, the contrast agent can be chosen from a drop-down menu.

Future procedural integration into routine CT acquisition is conceivable by on-site integration into the scanning process. After scout acquisition, the imaging data is routed to the local application, where the model predicts body weight within about one second on standard CPU hardware. The user selects the intended contrast agent, and the application converts predicted weight into a suggested contrast volume based on manufacturer-provided or user-defined conversion factors. Lastly, the radiographer verifies plausibility (particularly at extremes or in visually atypical cases) and can override the suggestion if needed. The predicted weight and resulting dose can be recorded to support traceability, auditing, and protocol standardization.

Several limitations should be considered. First, this was a single-center retrospective study with scanners and protocols from one vendor environment; external generalizability to other vendors, scout geometries, and clinical workflows remains to be proven. Second, abdominal-only scouts were underrepresented, and although performance remained good, estimates in this subgroup should be interpreted cautiously until confirmed in larger samples. Third, our cohort excluded patients under 18 years of age, so pediatric generalization is not supported. Fourth, body weight is only one determinant of optimal enhancement: physiology (cardiac output), venous access, injection rate, iodine concentration, scan timing, and clinical indication all influence contrast dynamics. Therefore, the presented tool should be understood as improving one important input (weight) rather than fully automating contrast protocol selection. Finally, we could not quantify off-centering and other acquisition metadata retrospectively, which may have contributed to residual error.

Future research encompassing the assessment of the performance of full-body scouts, validation using human tissue-equivalent phantoms, or an upper-bound performance evaluation might allow for further refinement of the proposed approach.

## Conclusion

Our study demonstrates a deep learning-based workflow for estimating contrast agent dosage from CT scout scans applicable to clinical routine. With a mean absolute error below 4 kg in weight estimation and interpretability analysis confirming plausible trustworthy predictive features, the approach shows substantial potential for clinical utility. The browser-based interface enables practical implementation in hospital settings while maintaining data privacy. Future work should validate this approach across larger cohorts and multiple clinical centers.

## Data Availability

The UI is publicly available at [https://tinyurl.com/ct-scout-weight] as stated in the manuscript. UI and training code is available under [https://www.osf.io/y2pzc/?view\_only=cfdd74947e4845528d7b66ca58b01f9a]. Training data is available upon reasonable research request to comply with our data regulations regarding sensitive clinical data.
